# Modulation of Intestinal Smooth Muscle Cell Function by BL-99 Postbiotics in Functional Constipation

**DOI:** 10.3390/foods14193441

**Published:** 2025-10-08

**Authors:** Wen Zhao, Mingkun Liu, Hanglian Lan, Ran Wang, Wei-Lian Hung, Jian He, Bing Fang

**Affiliations:** 1 Key Laboratory of Functional Dairy, Department of Nutrition and Health, China Agricultural University, Beijing 100190, China; 13614317400@163.com (W.Z.); wangran@cau.edu.cn (R.W.); 2 School of Food and Biological Engineering, Hefei University of Technology, Hefei 230009, China; liumingkun0203@163.com; 3 National Center of Technology Innovation for Dairy, Hohhot 100118, China; mindylan678@gmail.com (H.L.); hongweilian@nctid.cn (W.-L.H.); hejian@nctid.cn (J.H.)

**Keywords:** functional constipation, postbiotics, *Bifidobacterium animalis* subsp *lactis* BL-99, smooth muscle cells, glutamate metabolism

## Abstract

Postbiotics, as a novel class of functional components, have garnered considerable scholarly and industrial interest due to their distinctive advantages in food processing applications and their positive impact on human health. Although postbiotics have demonstrated potential in alleviating constipation, their specific mechanism of action and bioactive components remain unclear. This study aimed to investigate the ameliorative effects and potential mechanisms of postbiotics derived from *Bifidobacterium animalis subsp. lactis* BL-99 (BL-99) on FC using both in vivo and in vitro models. The findings revealed that both BL-99 and its postbiotics significantly mitigated FC symptoms, as evidenced by enhanced intestinal motility, and elevated fecal water content. Additionally, treatment with BL-99 postbiotics was associated with an increase in the thickness of the intestinal muscular layer and a reduction in apoptosis of intestinal smooth muscle cells (SMCs). Mechanistically, BL-99 postbiotics were found to enhance the contractile response and promote the proliferation of intestinal SMCs. Furthermore, untargeted metabolomics analysis identified two key bioactive peptides, Glu-Val and Glu-Leu, as the active components in BL-99 responsible for regulating SMC function. Collectively, these findings highlight the potential of BL-99 postbiotics as a promising functional food ingredient for alleviating FC, providing a novel and effective strategy for the developing dietary interventions targeting this condition.

## 1. Introduction

Functional constipation (FC) is a common gastrointestinal disorder characterized by difficulties in defecation, decreased frequency of bowel movements, hard stools, and a sensation of incomplete evacuation [1]. Epidemiological studies suggest that the global prevalence of FC is approximately 14%, with rates surpassing 20% in elderly populations [2,3]. FC not only significantly diminishes patients’ quality of life but is also associated with an increased risk of chronic diseases such as colorectal cancer [4,5], cardiovascular diseases [6], and Alzheimer’s disease [7]. As modern lifestyles become more fast-paced and the population continues to age, the incidence of FC is rising, underscoring the urgent need to develop safer and more effective intervention strategies.

Probiotics, which are active microorganisms that provide health benefits to the host, have been extensively studied for their potential to alleviate FC [8,9]. Research indicates that, compared to healthy individuals, adults with constipation exhibit an average reduction of 1 log10/g in *Bifidobacterium* and 1.4 log10/g in *Lactobacillus* in their fecal samples [10]. Notably, exogenous supplementation with probiotics can partially reverse the microbial dysbiosis and improve intestinal function.

A systematic review and meta-analysis of 15 randomized controlled trials demonstrated that specific probiotic strains significantly alleviate functional constipation (FC) symptoms in adults. This included a reduction in colonic transit time by 12 h, an increase in stool frequency by 1.5 stools per week, and a mitigation of symptoms associated with constipation [11]. Mechanistically, probiotics may exert their beneficial effects on FC through interactions with the enteric nervous system (ENS) [12], the promotion of neurotransmitter release such as serotonin (5-HT) and acetylcholine (Ach) [13,14], the enhancement of intestinal mucosal barrier function [15], and the modulation of intestinal metabolites such as short-chain fatty acids (SCFAs) [16,17] and tryptophan-related metabolites [13]. Furthermore, recent studies have identified that *Bifidobacterium longum* strains possessing the arabinogalactan utilization gene cluster *abf*A exhibit significant efficacy in alleviating constipation symptoms [18].

However, research indicates that the efficacy of probiotics in managing FC is not exclusively dependent on live bacteria; specific postbiotics also demonstrate the capability to alleviate constipation [19,20]. Postbiotics are bioactive preparations composed of inactivated microorganisms and their metabolites [21]. Unlike probiotics, postbiotics are capable of exerting health benefits without requiring the viability or metabolic activity of live microbial strains, making them suitable for addition to ambient-temperature food carriers where strain survival might be compromised [22]. Additionally, compared to live bacteria, postbiotics are more stable and safe during production and storage. These characteristics make postbiotics a promising new ingredient for driving the development of the functional food industry [23]. A randomized, controlled, double-blind clinical trial demonstrated that a two-week daily intake of 25 billion CFU of heat-inactivated *Bifidobacterium longum* CLA 8013 significantly increased bowel movement frequency, reduced defecation-related tension, and alleviated symptoms such as pain during defecation [19]. Ma et al. [20]. reported that a three-week postbiotic intervention, as evidenced by combined human and mouse experiments, markedly improved constipation symptoms, reduced defecation difficulty, and lowered anxiety scores. Although emerging evidence supports the potential of postbiotics in alleviating constipation, the underlying mechanisms of action and the identity of key bioactive components remain insufficiently elucidated.

*Bifidobacterium animalis* subsp. *lactis* BL-99 (BL-99), isolated from the intestines of healthy infants, has been shown in previous studies to alleviate constipation in its live bacterial form [24,25]. However, the functions and mechanisms of BL-99 postbiotics remain inadequately understood. In this study, a loperamide (LOP)-induced constipation mouse model was employed to systematically evaluate the effects of BL-99 postbiotics on alleviating FC, including assessments of defecation parameters and intestinal tissue morphology. Subsequently, by utilizing mouse models and in vitro experiments, in conjunction with untargeted metabolomics analysis, the specific mechanisms and key bioactive components of BL-99 postbiotics on Smooth Muscle Cells (SMCs) were investigated. This study aimed to provide a theoretical basis for the precise application of postbiotics in intestinal motility disorders and to develop novel strategies for the microecological therapy of FC.

## 2. Materials and Methods

### 2.1. Bacterial Strains Cultivation and Preparing CFS Samples

*Bifidobacterium animalis* subsp. *lactis* BL-99 (BL-99) was supplied by Inner Mongolia Dairy Technology Research Center (Hohhot, China). The BL-99 strain was cultured in MRS Broth at 37 °C overnight prior to harvesting. The overnight cultures of BL-99 were harvested via centrifugation at 4500× *g* for 10 min, followed by three washes and resuspension in PBS to achieve a concentration of 1 × 10^10^ CFU/mL. For postbiotic preparation, the heat-killed bacteria were adjusted to concentrations of 1 × 10^7^ CFU/mL, 1 × 10^8^ CFU/mL, and 1 × 10^9^ CFU/mL, and subsequently inactivated at 121 °C for 10 min. The cell-free supernatant (CFS), serving as the postbiotic, was obtained by centrifugation at 10,000 × g for 10 min at 4 °C and filtered through a 0.22 μm sterile aqueous filter membrane.

### 2.2. Experimental Animals and Design

This study was approved by the Experimental Animal Ethics Committee of China Agricultural University (AW72305202-4-3), and performed in accordance with the Laboratory animal—Guidelines for ethical review of animal welfare. (GB/T 35892-2018, China) [26]. Eighty-four six-week-old male BALB/C mice, weighing between 18–22 g, were procured from Beijing Sibef Biotechnology Co., Ltd. (Beijing, China, Animal Use License No.: SCXK (Jing) 2024-0001). The rats were housed under controlled conditions with a temperature of 20  ±  2 °C, humidity of 55  ±  5%, and a 12-h light/dark cycle.

Following a one-week period of adaptive feeding, the mice were randomly assigned to seven distinct groups (n = 12 per group): the control group (Control), the loperamide-induced constipation model group (Model), the lactulose-positive control group (Positive), high-dose (H_BL-99: 1 × 10^9^ CFU/mL) and low-dose (L_BL-99: 1 × 10^8^ CFU/mL) groups of BL-99 live bacteria, and high-dose (H_CFS: 1 × 10^9^ CFU/mL) and low-dose (L_CFS: 1 × 10^8^ CFU/mL) groups of BL-99 CFS. The experimental duration was 28 days, comprising a 7-day modeling phase and a 21-day treatment phase. During the modeling phase, all groups except the Control group, which received 0.9% NaCl solution via gavage, were administered 1.5% loperamide (0.1 mL daily) to induce constipation. Successful modeling was determined by a significant delay in the time to first red stool excretion in the Model group compared to the Control group, along with statistically significant differences in fecal water content and the number of fecal pellets over a 5-h period (*p* < 0.05). During the treatment phase (days 8–28), each intervention group received specific treatments: the Positive group received 0.1 mL of lactulose (67%), while other groups received 0.1 mL of BL-99 live bacteria or CFS. At the end of the experiment, the animals were sacrificed, and the colonic tissues were collected aseptically, and stored at −80 °C.

### 2.3. Fecal Parameter Measurement

The time to first red stool was measured on day 28. The procedure was as follows: mice were fasted for 12 h (water ad libitum) before oral gavage of 0.1 mL 15% carmine red solution. Animals were individually housed in sterile cages without bedding, and the time to first red fecal expulsion was recorded along with 5-h fecal pellet counts. For intestinal transit rate calculation, mice were euthanized 30 min post-gavage; the entire intestine was excised to measure total length (S1) and carmine migration distance (S2). The intestinal transit rate is calculated according to the following formula:

Intestinal transit rate (%) = S2/S1 × 100%(1)

Intestinal tissues were dehydrated, embedded, and sectioned for hematoxylin and eosin (H&E) staining to assess colon histological changes.

The experimenter who handled the mice and conducting experiments related to fecal parameters was blinded to the group allocation of the animals throughout the experiment and data analysis.

### 2.4. Colon Tissue TUNEL Staining

According to the manufacturer’s instructions, the TUNEL (Terminal Deoxynucleotidyl Transferase dUTP Nick-End Labeling) BrightRed Apoptosis Detection Kit (Vazyme, Nanjing, China, A113) was used to detect cell apoptosis in colon tissue sections. Briefly, the colon tissues were sectioned and incubated with proteinase K (20 μg/mL) at room temperature for 20 min, followed by three washes with PBS. The sections were then incubated with 50 μL of TUNEL reaction mixture was added to each sample. After incubation at 37 °C for 1 h, the sections were washed three times with PBS. Subsequently, the sections were counterstained with DAPI for 5 min. Images were acquired using a confocal microscope (Zeiss, Germany, Meta Zeiss LSM 780, objective WI 63X).

### 2.5. Transcriptomic Analysis

Total RNA was extracted from mouse intestinal tissues using Trizol reagent (Invitrogen, Waltham, MA, USA). RNA-Seq library preparation, sequencing, and primary bioinformatic analysis were performed by Shanghai Majorbio Bio-pharm Technology Co., Ltd. (Shanghai, China). Briefly, polyA-enriched mRNA libraries were constructed and sequenced on an Illumina platform. Differentially expressed genes were then identified using the edgeR package (version 3.12.1), applying a threshold of |log2 (fold change)| ≥ 1 and a *p*-value < 0.05. Finally, KEGG pathway enrichment analysis for these genes was performed with KOBAS software (Version 2.0, https://cyverse.atlassian.net/wiki/spaces/TUT/pages/258736176/KOBAS, accessed on 1 February 2025), using a significance cutoff of *p* < 0.05.

### 2.6. HAVSMC Culture

The human aortic vascular smooth muscle (HAVSMCs, Otwo Biotech, Shenzhen, China) cells were placed in the smooth muscle cell medium culture medium and then cultured in a cell culture box at 37 °C. The culture conditions in the box were 5% CO_2_, 95% air and 100% humidity. When the cell adhesion area reached approximately 80%, the subsequent experiments were conducted. The CCK-8 method was used to detect the effects of different intervention groups on the viability of HAVSMCs.

### 2.7. Untargeted Metabolomics Analysis

Untargeted metabolomics was performed to profile metabolites in the cell-free supernatant. Sample preparation followed our previous research [27]. LC-MS/MS analysis was performed using the Thermo UHPLC-Q Exactive HF-X mass spectrometer (Thermo Fisher Scientific, Swedesboro, NJ, USA). The cell-free supernatant was homogenized in methanol/water (4:1) containing internal standards. After centrifugation of the samples, the supernatant was taken for analysis. Chromatographic separation was carried out using an ACQUITY UPLC HSS T3 column, with a gradient elution using water and acetonitrile (both containing 0.1% formic acid). Mass spectrometric analysis in positive and negative ion modes was performed using the Q Exactive HF-X mass spectrometer.

### 2.8. Quantification of Gene Expression by Real-Time PCR

Extract total RNA from colon tissues and HAVSMCs using Trizol reagent. Synthesize cDNA from 1 µg of total RNA using PrimeScript RT kit (Takara, Japan). qPCR was performed on a LightCycler 480 system using SYBR Green Master Mix. The base sequences of the target genes (*Ip3r*, *Calm*, *Mlc*, *Mlck*, *Cdk1*, *Cdc25c*, *Plk1*, *Wee1*) were designed with Primer Premier 5.0 software (Table 1). With *gapdh* used as the reference gene, relative expression levels were calculated using the 2^−ΔΔCT^ method [28].

### 2.9. Statistical Analysis

The data are presented as mean ± standard error. All experiments were repeated at least three times. The normality of all data was confirmed using the Shapiro–Wilk test, and the homogeneity of variances was verified using Levene’s test. Data that met these assumptions were analyzed using parametric tests. To conduct inter-group comparisons, a two-tailed Student’s *t*-test and Dunnett’s multiple comparison test were used for analysis of variance (ANOVA). Data analysis was performed using GraphPad Prism 10. A *p*-value < 0.05 indicates a significant difference, and a *p*-value < 0.01 indicates an extremely significant difference.

## 3. Results

### 3.1. BL-99 and Its Postbiotics Enhanced the Intestinal Motility of Constipated Mice

**Figure 1:**
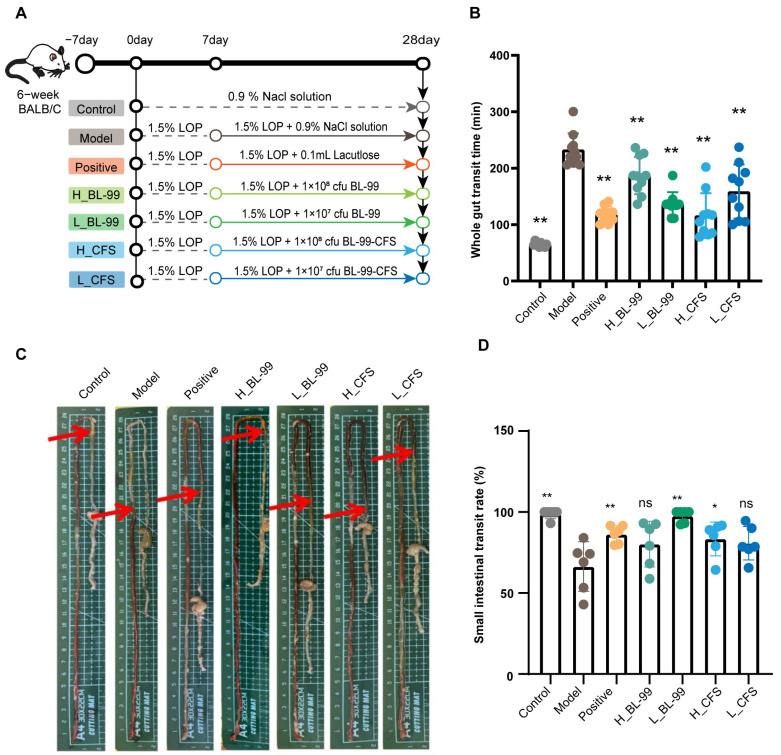
BL-99 live bacteria and postbiotics enhanced the intestinal motility in constipated mice. (**A**) Experimental design schematic diagram; (**B**) time of the first defecation with red stool (n = 12 mice/group); (**C**,**D**) intestinal transit rate (n = 6 mice/group; arrows, carmine migration distance). The data represent the mean ± standard deviation. Statistical significance was determined by one-way ANOVA followed by LSD post-hoc test for multiple comparisons. * *p* < 0.05, ** *p* < 0.01 versus the Model group, “ns” indicates no statistical difference.

To assess the efficacy of BL-99 and its postbiotics on FC, mice were initially treated with LOP to induce constipation. Subsequently, they received administrations of live BL-99 bacteria and CFSs over a period of three weeks (Figure 1A). The results indicated that, relative to the Control group, LOP treatment significantly extended the time to first defecation in mice (Control: 64.9 ± 3.67 min vs. Model: 232.6 ± 27.59 min, *p* < 0.01). However, interventions with both live BL-99 bacteria and BL-99 CFS significantly reduced the time to first defecation. Notably, the groups receiving low-dose BL-99 (L_BL-99,136.6 ± 19.86 min) and high-dose CFS (H_CFS,115 ± 37.71 min) demonstrated the most substantial effects, with results approaching those observed in the Positive control group (181.1 ± 13.01 min) (Figure 1B). The data for small intestine transit rate are presented in Figure 1C. In the Model group, the intestinal transit rate of mice was significantly decreased (Control: 98.83 ± 2.86% vs. Model: 66.35 ± 13.96%, *p* < 0.01). In contrast, compared to the Model group, the intestinal transit rates in the L_BL-99 and H_CFS groups increased to 97.55 ± 3.80% and 83.38 ± 10.43%, respectively (*p* < 0.01), exhibiting improvements similar to those in the Positive control group (86.22 ± 4.85%). These findings suggest that both L_BL-99 and H_CFS treatments effectively enhance intestinal motility in mice with induced constipation.

### 3.2. BL-99 and Its Postbiotics Improved Constipation Related Symptoms in Mice

**Figure 2:**
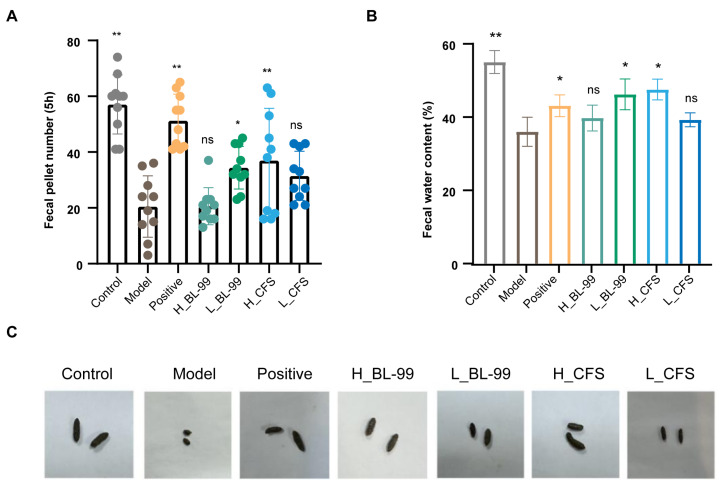
High and low doses of BL-99 live bacteria and postbiotics improved constipation related indicators in mice. (**A**) Number of fecal pellets within 5 h; (**B**) fecal moisture content; (**C**) fecal morphology. The data represent the mean ± standard deviation (n = 12/group). Comparisons were made using one-way ANOVA analysis and LSD post-hoc test. * *p* < 0.05, ** *p* < 0.01, “ns” indicates no statistical difference.

The study investigated constipation-related symptoms in mice, revealing that the Model group exhibited a significant reduction in the number of fecal pellets within a 5-h period compared to the control group (20.5 ± 10.46 vs 57.1 ± 10.07 pellets/5 h, *p* < 0.01), indicative of typical constipation symptoms. In contrast, treatment with the L_BL-99 (34.4 ± 7.27 pellets/5 h) and H_CFS (37 ± 17.76 pellets/5 h) groups significantly increased the number of fecal pellets within the same timeframe compared to the Model group (*p* < 0.05) (Figure 2A). Fecal water content, a crucial indicator for evaluating constipation improvement, was notably decreased in mice following loperamide induction compared to the control group (36 ± 3.97% vs. 55.1 ± 3.15%, *p* < 0.01). However, supplementation with BL-99 in each group partially reversed this decline, with the L_BL-99 (46.23 ± 4.21%) and H_CFS (47.54 ± 2.81%) groups showing particularly significant effects (*p* < 0.05), resulting in fecal moisture levels nearing those of the Positive group (43.1 ± 2.97%) (Figure 2B). Visual comparison of fecal images indicated that mice in all BL-99 groups and the Positive group produced feces with a more slender and softer texture, whereas feces from the Model group exhibited a dry, granular appearance (Figure 2C). These results indicate that the L_BL-99 and H_CFS interventions can effectively alleviate the constipation symptoms in mice.

### 3.3. BL-99 and Its Postbiotics Promoted the Thickening of the Intestinal Muscle Layer in Constipated Mice

**Figure 3:**
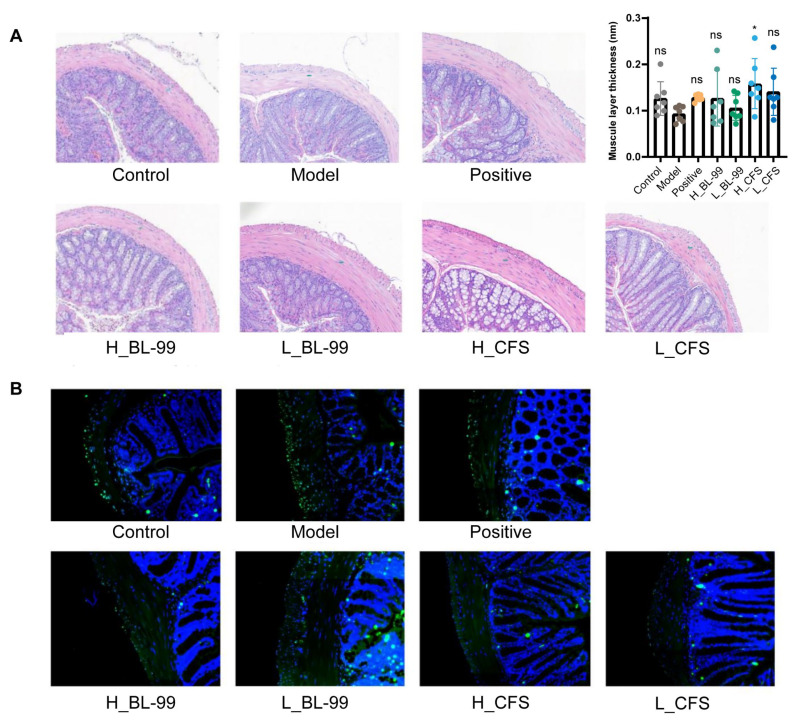
The effect of high and low doses of BL-99 live bacteria and postbiotics on intestinal smooth muscle layer of mice. (**A**) Morphology of colon tissue and thickness of intestinal muscular layer in mice; (**B**) The distribution of apoptotic cells (green) in the colonic smooth muscle tissue was observed using Tunel, and DAPI (blue) was used for staining as a control; data represent the mean ± standard deviation (n = 6/group). Comparisons were made using one-way ANOVA analysis and LSD post hoc test. * *p* < 0.05, “ns” indicates no statistical difference.

Smooth muscle serves as a crucial effector organ in the regulation of intestinal motility. In this study, HE staining was employed to examine the colonic tissue of mice, with particular emphasis on alterations in the thickness of the smooth muscle layer. Following the induction of LOP, a noticeable thinning of the intestinal muscle layer was observed in comparison to the Control group, along with the emergence of a gap between the muscle layer and the submucosal layer. These morphological changes may be associated with diminished intestinal peristaltic function and defecatory difficulties (Figure 3A,B). Intervention with BL-99 live bacteria and CFSs resulted in an increase in the thickness of the intestinal muscle layer in mice, with the H_CFS group exhibiting the most pronounced effect (*p* < 0.05, Figure 3A,B). Additionally, TUNEL staining was conducted on the colonic tissues to assess apoptosis in intestinal SMCs. The findings revealed an increase in apoptotic cells within the colonic muscular SMCs in the Model group compared to the Control group. However, treatment with BL-99 live bacteria and CFSs significantly reduced the number of apoptotic cells in these tissues (Figure 3A). These results indicate that the BL-99 CFS may exert the effect of alleviating FC by inhibiting the apoptosis of intestinal SMCs and maintaining the thickness of the colon muscle layer.

### 3.4. BL-99 and Its Postbiotics Enhanced the Contraction and Proliferation Functions of Intestinal SMCs

**Figure 4:**
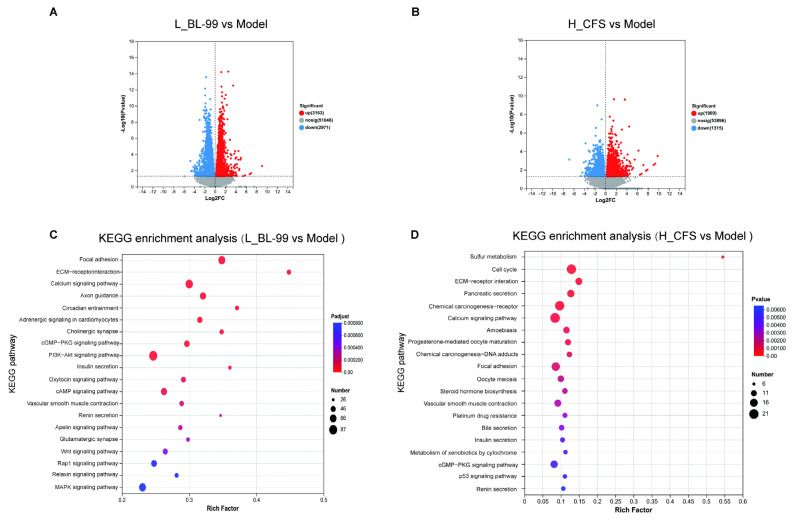
RNA-sq sequencing results of the colonic muscular layer in L_BL-99 and H_CFS groups of mice. (**A**) DEG volcano plot (L_BL-99 vs. Model); (**B**) DEG volcano plot (H_CFS vs. Model); (**C**) KEGG pathway enrichment analysis of differential genes (L_BL-99 vs. Model); (**D**) KEGG pathway enrichment analysis of differential genes (H_CFS vs. Model); The data represent the mean ± standard deviation (n = 4/group).

The experimental results indicated that the L_BL-99 and H_CFS groups exhibited the most pronounced improvement in alleviating constipation. To elucidate the molecular mechanisms underlying the effects of BL-99 and its postbiotics on constipation, RNA-Seq analysis was conducted on mice from the Model, L_BL-99, and H_CFS groups. To reduce interference from the mucosal layer, a 2-cm segment of the proximal colon was collected, with the epithelium and villi (non-muscle layers) removed prior to RNA-Seq and subsequent RT-PCR analyses. Differentially expressed genes (DEGs) were identified across groups using the criteria of *p* < 0.05 and |log2FC| ≥ 1. The findings revealed that, compared to the Model group, the L_BL-99 group exhibited 6134 significantly differentially expressed genes, including 3163 up-regulated and 2971 down-regulated genes (Figure 4A). Conversely, the H_CFS group showed 3265 significantly differentially expressed genes relative to the Model group, comprising 1961 up-regulated and 1304 down-regulated genes (Figure 4B). KEGG functional enrichment analysis demonstrated that the DEGs in both the L_BL-99 and H_CFS groups were predominantly enriched in pathways such as ECM-receptor interaction and calcium signaling, cGMP-PKG signaling pathway, PI3K-Akt signaling pathway and vascular smooth muscle contraction, which are all closely associated with SMC contraction and the cell proliferation cycle (Figure 4C,D).

**Figure 5:**
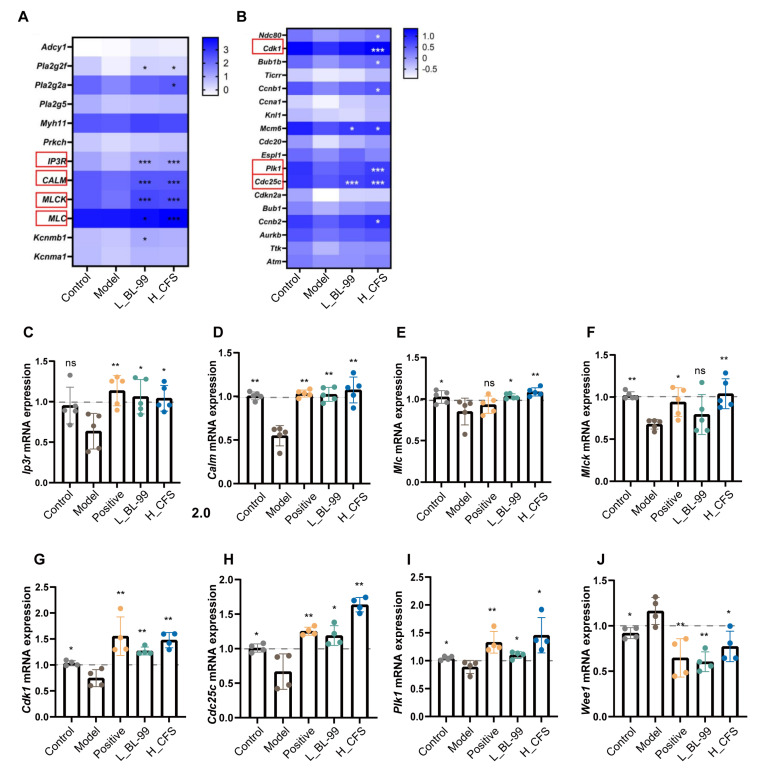
L_BL-99 and H_CFS promoted the contraction and proliferation functions of intestinal SMCs in constipated mice. (**A**) Clustering analysis of genes involved in the SMC contraction pathway, red frame, SMC contraction related genes; (**B**) clustering analysis of genes involved in the SMC cycle pathway, red frame, SMC proferation related genes; (**C**–**F**) mRNA expression of colon SMC contraction-related genes *Ip3r*, *Calm*, *Mlc* and *Mlck*; (**G**–**J**) mRNA expression of SMC proliferation-related genes *Cdk1*, *Cdc25c*, *Plk1* and *Wee1* in the colon. The data represent the mean ± standard deviation (n = 6/group). Comparisons were made using one-way ANOVA analysis and LSD post-hoc test. * *p* < 0.05, ** *p* < 0.01, *** *p* < 0.001, “ns” indicates no statistical difference.

Based on the results of the KEGG analysis, a cluster heatmap was generated for the genes associated with the contraction and proliferation functions of SMCs. The contraction of SMCs is contingent upon the intracellular calcium ion (Ca^2+^) concentration. Specifically, the accumulation of intracellular Ca^2+^ activates the downstream calmodulin (CALM)–myosin light chain kinase (MLCK) signaling cascade. The activation of MLCK enhances the phosphorylation of myosin light chain 20 (MLC20), facilitating actin–myosin cross-linking and ultimately inducing SMC contraction [29]. As illustrated in Figure 5A, both L_BL-99 and H_CFS treatments significantly upregulated the expression of *Ip3r*, *Calm*, *Mlc*, and *Mlck* genes compared to the Model group. The IP3R functions as a calcium ion channel protein located on the endoplasmic reticulum membrane, modulating the release of intracellular Ca^2+^ (*p* < 0.001). Furthermore, H_CFS treatment markedly increased the expression levels of *Cdk1*, *Cdc25c*, and *Plk1* genes, which are associated with the cell proliferation cycle (Figure 5B, *p* < 0.001).

To corroborate the transcriptome findings, quantitative reverse transcription PCR (qRT-PCR) analysis was conducted to assess the expression of genes related to SMC contraction and proliferation. The PCR analysis demonstrated that, in comparison to the Model group, both the L_BL-99 and H_CFS groups significantly upregulated the mRNA expression of *Ip3r*, *Calm*, *Mlc*, and *Mlck*, which are integral to SMC contraction (Figure 5C–F, *p* < 0.05). Furthermore, these groups exhibited increased mRNA expression levels of *Cdk1*, *Cdc25c*, and *Plk1*, which are essential for SMC proliferation, while concurrently downregulating the expression of *Wee1*, a known negative regulator of proliferation (Figure 5G–J, *p* < 0.05). These observations were corroborated by the RNA-seq data. These results imply that L_BL-99 and H_CFS may enhance intestinal muscle function by facilitating the contraction of intestinal SMCs and augmenting their proliferative capacity.

### 3.5. BL-99 Postbiotics Regulated the Contraction and Proliferation Functions of SMCs In Vitro

**Figure 6:**
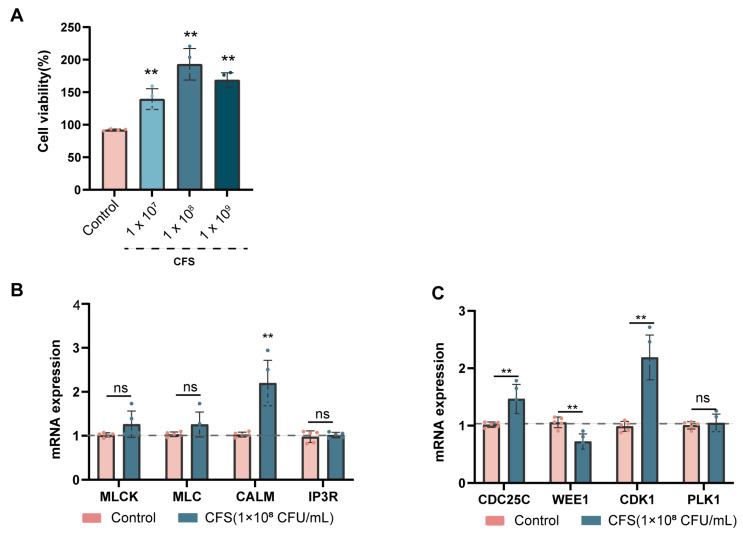
BL-99 postbiotics modulate the expression of genes regulating contractile function of SMCs in vitro. (**A**) Effects of different concentrations of BL-99 CFS (1 × 10^7^, 1 × 10^8^, 1 × 10^9^ CFU/mL) on the viability of HVSMCs; (**B**) effects of BL-99 CFS on the relative mRNA expression levels of genes involved in the SMCs contraction: *MLCK, MLC, CALM, IP3R*; (**C**) effects of BL-99 CFS on the relative mRNA expression levels of genes involved in the SMC cycle: *CDC25C, WEE1, CDK1, PLK1*. The data represent the mean ± standard deviation from three independent biological replicates (n = 3/group). Comparisons were made using one-way ANOVA analysis and LSD post-hoc test. ** *p* < 0.01 versus the control group, “ns” indicates no statistical difference.

The outcomes of the animal experiments indicate that BL-99 postbiotics could mitigate constipation by modulating intestinal SMC function, with efficacy surpassing that of the live bacteria group. To substantiate these findings, in vitro experiments were conducted using human aortic vascular smooth muscle cells (HAVSMCs) treated with BL-99 CFSs. The experiment incorporated a control group and intervention groups exposed to varying concentrations of BL-99 CFSs (10^7^ CFU/mL, 10^8^ CFU/mL and 10^9^ CFU/mL). The findings demonstrated that, relative to the control group, treatment with different concentrations of BL-99 CFSs significantly enhanced the viability of HAVSMCs (*p* < 0.01, Figure 6A). Notably, the treatment group at 10^8^ CFU/mL exhibited the most pronounced effect, leading to the selection of this concentration for subsequent experiments.

The qRT-PCR analysis indicated that, in comparison to the control group, treatment with BL-99 CFSs significantly upregulated the mRNA expression level of the *CALM* gene by 2.14-fold (*p* < 0.01, Figure 6B), suggesting an increased sensitivity of SMCs to Ca^2+^, which may enhance their contractile function. Furthermore, the mRNA expression levels of the key cell cycle regulators *CDC25C* and *CDK1* were significantly elevated by 1.44-fold and 2.22-fold, respectively (*p* < 0.01), while the mRNA expression level of *WEE1* was significantly reduced to 0.68-fold that of the control group (*p* < 0.01, Figure 6C). These observations are consistent with the in vivo experimental results. However, there was no significant difference in the mRNA expression levels of the *IP3R*, *MLC*, *MLCK*, and *PLK1* genes in the postbiotic-treated groups compared to the control group (*p* > 0.05, Figure 6B,C). Collectively, the cell experiments further validated that BL-99 CFSs enhance the viability of SMCs, promote SMC contraction and facilitate cell cycle progression.

### 3.6. Glu-Val and Glu-Leu Are Key Bioactive Components of BL-99 Postbiotics

**Figure 7:**
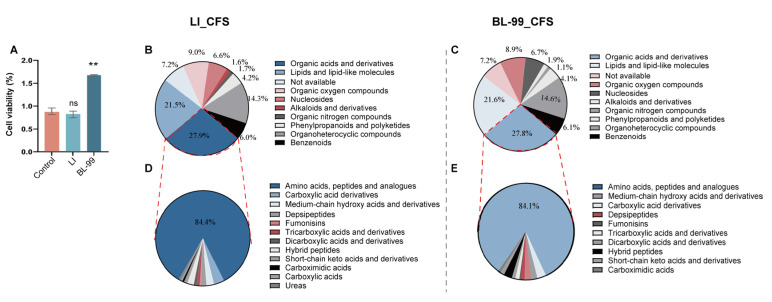

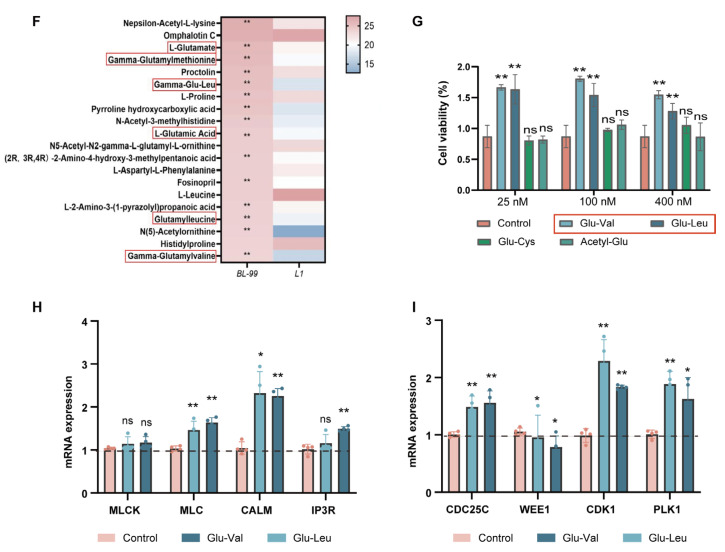
Glu-Val and Glu-Leu modulate the expression of genes regulating contractile function of SMCs in vitro. (**A**) Effects of BL-99 and L1 CFS on HVSMC viability; (**B**) secondary classification of L1 metabolites; (**C**) secondary classification of BL-99 metabolites; (**D**) quaternary classification of L1 metabolites; (**E**) quaternary classification of BL-99 metabolites; (**F**) cluster analysis of differential amino acid metabolites between L1 and BL-99, red frame, glutamic acid and its metabolites; (**G**) effects of different glutamate metabolites on HAVSMC viability; (**H**,**I**) relative mRNA expression levels of genes related to (**H**) SMC contraction and (**I**) cell cycle progression after treatment with Glu-Val and Glu-Leu; the data represent the mean ± standard deviation from three independent biological replicates (n = 3/group). Comparisons were made using one-way ANOVA analysis and LSD post-hoc test. * *p* < 0.05, ** *p* < 0.01 versus the control group, “ns” indicates no statistical difference.

To elucidate the primary components of BL-99 CFSs that contributed to the alleviation of FC, we conducted an untargeted metabolomics analysis on the BL-99 CFS. For this study, CFS of *Lactobacillus rhamnosus* L1 (L1), which exhibited no proliferative activity on HAVSMCs (Figure 7A), served as a negative control. Comparative analyses indicated that organic acids and their derivatives constituted the largest proportion in the CFS of both L1 (27.9%) and BL-99 (27.8%) (Figure 7B,C). Further categorization within the organic acids revealed that amino acids represented the most substantial proportion, with BL-99 at 84.1% and L1 at 84.4% (Figure 7D,E). A comparison of the top 20 amino acid compounds in the BL-99 CFS with those in L1 identified six metabolites related to glutamic acid (Figure 7F). These findings suggested that glutamic acid and its metabolites may play a pivotal role in the differential functional effects of BL-99 and L1 on constipation relief.

Based on the untargeted metabolomics results, four metabolites including glutamic acid dipeptides (Glu-Val, Glu-Leu and Glu-Cys) and acetyl-glutamic acid (Acetyl-Glu) were selected for further investigation. HAVSMCs were treated with these metabolites at concentrations of 25 nM, 100 nM, and 400 nM, and cell viability was assessed. The results showed that Glu-Val and Glu-Leu significantly enhanced cell viability (*p* < 0.01), whereas Glu-Cys and Acetyl-Glu did not exhibit such effects (Figure 7G).

qRT-PCR analysis further revealed that compared to the control group, Glu-Val and Glu-Leu significantly upregulated the mRNA levels of *MLC* and *CALM* (*p* < 0.05, Figure 7H), as well as the mRNA levels of cell cycle-related genes *CDC25C*, *CDK1* and *PLK1* (*p* < 0.05). Additionally, the mRNA expression level of *WEE1* was significantly downregulated (*p* < 0.05, Figure 7I). These findings suggest that Glu-Val and Glu-Leu were the key active components of BL-99 postbiotics that enhance the function of SMCs.

## 4. Discussion

FC is a prevalent gastrointestinal disorder that significantly diminishes patients’ quality of life and poses a considerable medical burden. Recent evidence suggests that postbiotics may have a beneficial impact on intestinal health. Nevertheless, the specific mechanisms of action and active components of postbiotics remain largely undefined. This study demonstrated that BL-99 postbiotics, along with their key active components Glu-Val and Glu-Leu, enhanced the contractility of intestinal SMCs, facilitated the cell cycle to preserve the muscular layer’s thickness, and effectively alleviated FC symptoms in mice. The research elucidated the molecular mechanisms by which postbiotics regulate intestinal motility and offered novel insights for developing constipation treatment strategies that target SMC functions.

As a novel functional ingredient, postbiotics exhibit significant potential for food industry applications, owing to their high safety, stability, and compatibility with sustainable development principles. Research indicates that postbiotics retain 90–95% of their bioactivity after ultra-high-temperature pasteurization, and are more easily metabolized and absorbed by the body, exhibiting advantages in multi-organ bioavailability [30]. Nevertheless, the precise health benefits and underlying mechanisms of postbiotics remain insufficiently explored. Our research findings indicated that the postbiotics derived from BL-99 can significantly mitigate symptoms of LOP-induced constipation in mice. This includes a reduction in the time to first defecation, an increase in intestinal transit rate, fecal output volume, and fecal moisture content. Consistent with our findings, Zhang et al. [31] reported that postbiotics from *Lactobacillus casei* PE0401 can modulate the composition of intestinal microbiota and enhance SCFA levels, thereby alleviating LOP-induced constipation symptoms in mice. Furthermore, recent clinical trials have demonstrated that heat-treated fermented milk containing *Lactobacillus helveticus* CP790 can improve fecal consistency and reduce straining during defecation in healthy adults [32]. Additionally, the postbiotic Probiotic-Eco has been shown to significantly alleviate constipation symptoms, reduce defecation effort, and lower anxiety scores in patients with functional constipation [20]. These findings collectively suggest that postbiotics hold promise as functional food strategy for the management of functional constipation.

Intestinal dysmotility is a fundamental physiological and pathological feature of FC [33]. The regulatory mechanisms underlying this condition encompass the contractile function of intestinal SMCs, the activity of the ENS, the metabolism of gut microbiota, and their intricate interactions [33]. Among these factors, intestinal SMCs act as the primary effectors of intestinal motility, with their rhythmic contractions and relaxations exerting a direct impact on the efficiency of intestinal transit [34]. Despite this, current therapeutic strategies for FC predominantly target the modulation of the ENS [35,36] or the manipulation of gut microbiota [37,38,39], with insufficient attention given to the function of intestinal SMCs.

In our study, we observed that a LOP-induced constipation mouse model exhibited thinning of the intestinal muscular layer and increased apoptosis of SMCs. Intervention with BL-99 postbiotics significantly ameliorated these pathological alterations. Specifically, the muscular layer thickness was restored to levels comparable to the control group, and apoptosis of SMCs was reduced. Mechanistically, BL-99 was found to upregulate IP3R-mediated calcium release, thereby activating the CALM-MLCK signaling pathway and promoting the recovery of SMC contractile function. Furthermore, this study revealed that BL-99 postbiotic treatment significantly upregulated the expression of cell cycle positive regulators PLK1 and CDC25C while inhibiting the activity of the negative regulator WEE1, ultimately facilitating the cell cycle in SMCs. Previous research has similarly documented the phenotypic effects of probiotic metabolites on intestinal smooth muscle; however, these studies often lack an exploration of the underlying mechanisms. Ammoscato et al. [40] reported that LGG supernatant conferred protection to lipopolysaccharide (LPS)-induced human intestinal smooth muscle cells from damage, with recovery of SMC shortening to 84.1% ± 4.7% and recovery of contraction inhibition to 85.5% ± 6.4%. Sobol et al. [41] demonstrated that metabolites from *Lactobacillus plantarum* enhanced the contraction response of rat colonic SMCs, which was accompanied by an increase in intracellular Ca^2+^ concentration. Dai et al. [42] indicated that the probiotic metabolite butyrate significantly induced the proliferation of HISMCs, characterized by accelerated cell cycle progression and increased DNA replication. Collectively, these results suggest that intestinal SMCs may serve as a significant target for postbiotic interventions.

Postbiotics are collectively defined as non-viable bacterial components and their metabolites [23]. Several studies have attempted to explore how different postbiotic components may alleviate constipation through various mechanisms. For example, probiotic-derived metabolites such as succinic acid, 3-indolepropionic acid, and 5-HT have been shown to exert anti-constipation effects by stimulating mucin-2 secretion and promoting anti-inflammatory responses [20]. SCFAs facilitated the release of 5-HT from enterochromaffin cells, thereby activating intestinal neurons and enhancing colonic motility [43]. Furthermore, bacterial components such as LPS can interact with Toll-like receptors on smooth muscle cells (SMCs), influencing intestinal contraction function [44]. In the present study, through untargeted metabolomics comparative analysis, we identified Glu-Val and Glu-Leu as key active dipeptides in BL-99 postbiotics that alleviate functional constipation symptoms by enhancing SMC function. However, Glu-Cys and Acetyl-Glu were ineffective. This might be due to their specific conformation, which is unfavorable for their binding to target receptors or enzymes, or for their absorption by cells. Previous research has demonstrated that glutamate not only serves as a crucial precursor of neurotransmitters but also regulates the contractile phenotype by activating metabotropic glutamate receptors in SMCs [45]. Meanwhile, research found that glutamate can enhance the contraction response of visceral SMCs by augmenting the activity of cholinergic efferent neurons [46]. Among them, Glu-Val has been confirmed to reduce the expression of pro-inflammatory cytokines and chemokines in the colon by activating calcium-sensing receptors (CaSRs), thereby alleviating intestinal injury [47,48]. In summary, these studies suggest that Glu-Val and Glu-Leu have the potential to serve as novel postbiotics markers for improving constipation.

Several limitations should be acknowledged. First, as a preclinical study, the improvement effect of BL-99 postbiotics requires validation in human clinical trials. Second, the mouse model of constipation may not fully recapitulate the complex pathophysiology of human chronic constipation, may limit the depth and breadth of mechanistic investigations. Third, the exclusive use of male mice precludes the evaluation of potential sex-based differences in response to treatment, highlighting the need for future studies to include both sexes.

## 5. Conclusions

This study investigated the effects of BL-99 postbiotics—a natural active ingredient—on constipation symptoms by enhancing intestinal smooth muscle function, specifically by promoting SMC contraction and proliferation. Crucially, Glu-Leu and Glu-Val were identified as the key bioactive components responsible for this regulatory effect. Our findings provide important mechanistic insights into how postbiotics modulate SMC function. Given their bioactivity in the gut stability and their stability during food processing, these findings underscore the potential of postbiotics as a valuable functional food strategy for addressing FC.

## Figures and Tables

**Table 1:** Primer list.

Gene Name	Primer Sequence/(5′-3′)(Mouse)	Primer Sequence/(5′-3′)(Human)
*Gapdh*	F: AAGCCCATCACCATCTTCCA R: CACCAGTAGACTCCACGACA	F: AGATCCCTCCAAAATCAAGTGG R: GGCAGAGATGATGACCCTTTT
*I* *p* *3* *r*	F: TGGCAGAGATGATCAGGGAAA R: GCTCGTTCTGTTCCCCTTCAG	F: GTGACAGGAAACATGCAGACTCG R: CAGCAGTTGCACAAAGACAGGC
*C* *alm*	F: ACTGGGTCAGAACCCAACAG R: GTTCTGCCGCACTGATGTAA	F: CCAACAGAAGCTGAATTGCAGGA R: CAAAGACTCGGAATGCCTCACG
*M* *lc*	F: GAGGGCTACGTCCAATGTCT R: GTCGTGCAGGTCCTCCTTAT	F: GAGGGCAAAGGACCCATTAAC R: CTTCTGGGTCCGTTCCATTAAG
*M* *lck*	F: GTTCATCAGCAAGCCTCGTT R: TTCTGGAGCAGCTCAAAGTG	F: GAGGTGCTTCAGAATGAGGACG R: GCATCAGTGACACCTGGCAACT
*C* *dk* *1*	F: GTCCGTCGTAACCTGTTGAG R: TGACTATATTTGGATGTCGAAG	F: GGAAACCAGGAAGCCTAGCATC R: GGATGATTCAGTGCCATTTTGCC
*C* *dc* *25* *c*	F: CATTCAGATGGAGGAGGAAGAG R: CACTGTGTCTGGGCTGATATAC	F: AGAAGCCCATCGTCCCTTTGGA R: GCAGGATACTGGTTCAGAGACC
*P* *lk* *1*	F: TGGGTGGACTATTCGGACAAG R: ACCCCCACACTGTTGTCACA	F: GCACAGTGTCAATGCCTCCAAG R: GCCGTACTTGTCCGAATAGTCC
*W* *ee* *1*	F: GAAACAAGACCTGCCAAAAGAA R: GCATCCATCTAACCTCTTCACAC	F: GATGTGCGACAGACTCCTCAAG R: CTGGCTTCCATGTCTTCACCAC

## Data Availability

The original contributions presented in the study are included in the article; further inquiries can be directed to the corresponding author.
